# Feeding Rainbow Trout with Different Types of Non-Starch Polysaccharides: Impacts on Serum Metabolome and Gut Microbiota

**DOI:** 10.3390/metabo12121167

**Published:** 2022-11-23

**Authors:** Hang Zhou, Yu Liu, Jiongting Fan, Huajing Huang, Junming Deng, Beiping Tan

**Affiliations:** 1 College of Fisheries, Guangdong Ocean University, Zhanjiang 524088, China; 2 Aquatic Animals Precision Nutrition and High Efficiency Feed Engineering Research Centre of Guangdong Province, Zhanjiang 524088, China; 3 Key Laboratory of Aquatic, Livestock and Poultry Feed Science and Technology in South China, Ministry of Agriculture, Zhanjiang 524088, China; 4 Laboratory of Aquatic Animal Nutrition and Feed, College of Fisheries, Guangdong Ocean University, Zhanjiang 524088, China

**Keywords:** non-starch polysaccharides, gut microbiota, serum metabolome, rainbow trout

## Abstract

A 70-day feeding trial investigated the effects of dietary inclusion of different types of non-starch polysaccharides (NSPs) on gut microbiota and serum metabolome of rainbow trout. Four practical feeds (42% crude protein, 17% crude lipid) were prepared with 8% insoluble NSP (INSP, cellulose), 16.8% soluble NSP (SNSP, composed of 1.12% β-glucan, 1.28% mannan, 4.8% arabinoxylan, and 9.6% pectin), 24.8% NSPs (8% INSP + 16.8% SNSP), or no NSPs inclusion, respectively. Dietary NSPs inclusion had no significant influence on the Shannon, Simpson, ACE, and Chao1 indices of gut microbiota but induced a significant increase in the abundance of *Pseudomonas aeruginosa* and *Photobacterium kishitanii*, and a decrease in Firmicutes and *Alistipes finegoldii.* Besides, dietary SNSP upregulated the carnitine synthesis metabolic pathway. Our data suggest that dietary NSPs are detrimental to gut microbiota homeostasis and the health of rainbow trout, and dietary SNSP exhibit a stronger ability to interfere with physiological metabolism of rainbow trout than INSP. Therefore, the physiological effects of dietary NSPs, especially SNSP, should be carefully considered when designing the commercial feed formulations of rainbow trout.

## 1. Introduction

Rainbow trout (*Oncorhynchus mykiss*) is popular among consumers for its flavor and rich nutritional value, and it is now widely farmed in China [1]. As a carnivorous fish, it has a high dietary protein requirement and limited tolerance to dietary non-starch polysaccharides (NSPs) [2,3,4]. Recently, limited fishmeal has forced an increasing use of plant-based ingredients in the diet of rainbow trout [5]. Therefore, this species may suffer from high dietary NSPs [6]. NSPs are one of the reasons for limiting the use of plant-based feed ingredients in aquafeeds since they are present in large amounts and can reduce digestive enzyme activity and nutrient digestibility, impair intestinal and hepatic morphology, thereby inhibiting fish growth [7,8,9,10]. Moreover, dietary NSPs are effective in altering the structure and composition of the intestinal flora, which, in turn, affects intestinal health [8]. Dietary NSPs supplementation has been shown to disrupt the intestinal health of yellow catfish (*Pelteobagrus fulvidraco*) [7,10], rainbow trout [6], largemouth bass (*Micropterus salmoides*) [8,9,11], turbot (*Scophthalmus maximus* L.) [12] and genetically improved farmed tilapia (*Oreochromis niloticus*) [13]. Additionally, several studies have shown that soluble NSPs (SNSP) exert a greater impairment impact on gut health than insoluble NSPs (INSP) [6,7,10,11,13]. This may be due to the inconsistency between INSP and SNSP on the gut microbiota thus affecting gut health [14]. However, no studies have reported whether dietary INSP and SNSP differentially regulate the intestinal flora of rainbow trout.

Metabolomics enables efficient qualitative and quantitative analysis of metabolites in biological samples [15,16]. Noteworthy, intestinal flora and diets are the determinants of serum metabolome. Changes in both diet and microbiome can effectively alter serum metabolite profiles [17]. Therefore, the serum metabolome is commonly used to assess the effects of dietary treatments [16,18]. Recently, serum metabolomics-based screening for active metabolites produced by the interaction of diet and gut flora contributed to the search for nutritional strategies to improve health status [19]. A metabolite profile based on metabolomics can contribute to understanding the differences in the physiological effects of dietary INSP and SNSP on fish, as well as provide data support for finding strategies to cope with the challenge of dietary NSPs. Hence, the present study investigates the effects of dietary INSP and SNSP on the intestinal flora and serum metabolome in rainbow trout.

## 2. Material and Methods

### 2.1. Feed Production, Fish and Management

Four practical feeds (42% crude protein, 17% crude lipid) were prepared with 8% INSP (cellulose), 16.8% SNSP (composed of 1.12% β-glucan, 1.28% mannan, 4.8% arabinoxylan, and 9.6% pectin), 24.8% NSPs (8% INSP + 16.8% SNSP) or no NSPs inclusion, respectively. Feed preparation work was done with reference to the method described in our previous study [20], air-dried and stored at −20 °C until use. Feed nutrient constitutions and formulas are shown in Supplementary Table S1.

A total of 360 individuals with an average body weight of 50.08 ± 0.18 g were divided into 12 aquariums (800 L), 30 fish per replicate. The feeding trial lasted 70 days, and fish were fed twice daily to satiation and the culture water temperature was kept at 16–18 °C and dissolved oxygen 7.8–9.2 mg/L, flow rate 60 L/min.

### 2.2. Sampling Works

Sampling was initiated after the fish had been fasted for 24 h, and the fish were anesthetized using eugenol (1:12,000) solution. Four fishes per aquarium were randomly selected for collecting serum samples according to the methods described by Deng et al. [6]. Subsequently, these fishes were dissected on a sterile bench to take intestine samples. Four mixed intestine samples were collected per group, with each mixed sample containing three intestines (one intestine per aquarium) and then quickly frozen in liquid nitrogen.

### 2.3. Serum Metabolome Analysis

Sample preparation: Serum samples were first defrosted at 4 °C, then 100 μL serum was taken out in a 1.5 mL EP tube mixed with 300 μL methanol (Merck, German) and 10 μL internal standard solution (DL-2-Chlorophenylalanine, 2.9 mg/mL) 30 s, and centrifuged at 4 °C, 12,000 rpm for 15 min. Finally, 200 μL supernatant solution was taken for the subsequent analysis using a Liquid Chromatography Mass Spectrometry system (LC-MS, Thermo). Briefly, the samples were first separated using a column at 40 °C at a flow rate of 0.3 mL/min (Hypergod C18, 100 × 4.6 mm 3 μm), followed by mass spectrometry analysis. Meanwhile, quality control (QC) was performed by a mixed serum sample for an equilibrium LC-MC system. The raw data obtained from the LC-MS assays were analyzed using Compound Discoverer 3.0 (Thermo) following the methods described by Zhang et al. [21].

### 2.4. Microbiome Analysis

Bacteria DNA was extracted by a commercial kit using the SDS method (Magen, Guangzhou, China), following a quality and purity test. Subsequently, the DNA samples were amplified and purified according to the formulation of Liu et al. [22], followed by library construction and sequencing of the cDNA. Sequencing work was carried out by Hiseq2500 PE250 platform (Illumina, San Diego, CA, USA), and sequencing data analysis work was carried out by Novogene Co., Ltd. (Beijing, China).

### 2.5. Statistical Analysis

Data in this trial about Observed-otus, Chao1, Shannon et al. were calculated using Qiime (Version 1.9.1), and one-way analysis of variance (SPSS 22.0 software) was used for analysis, and finally showed as means ± SEM. Alpha diversity index inter-group variance analysis was performed using R software (Version 2.15.3). The T-test and Wilcox test were chosen when only two groups were compared, and the Tukey test and the Wilcox test of the agricolae package were chosen if there were more than two groups. *p* < 0.05 represents a significant difference.

## 3. Results

### 3.1. Quality Control and PCA Analysis

**Figure 1:**
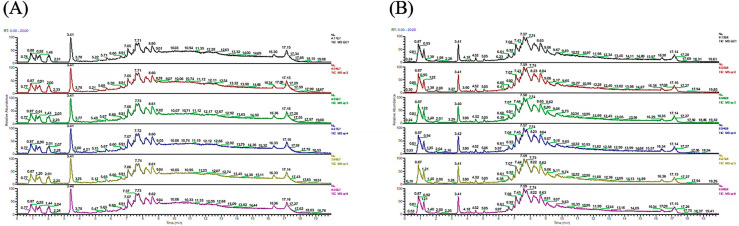
Metabolite profiles of quality control samples. (**A**) Positive ion mode, (**B**) negative ion model.

**Figure 2:**
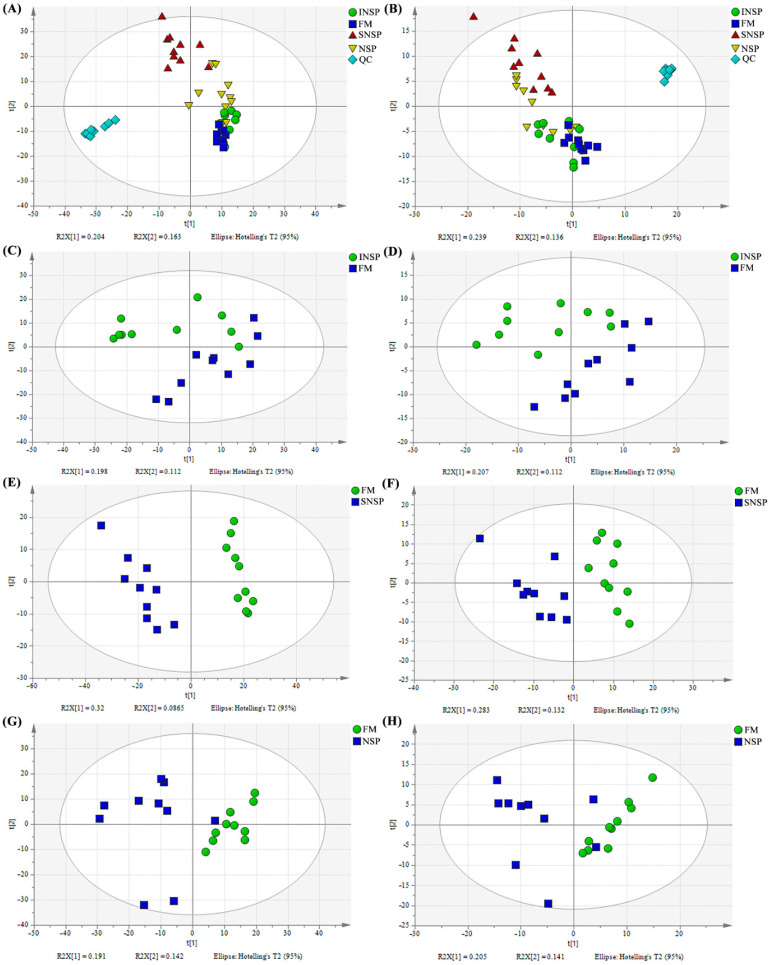
PCA results of serum metabolome profiles in rainbow trout fed with test diets. (**A**,**B**) All groups and QC samples in positive/negative ion model, respectively, (**C**,**D**) FM vs. CF in positive/negative ion model, respectively, (**E**,**F**) FM vs. NSP in positive/negative ion model, respectively, (**G**,**H**) FM VS NSP+CF in positive/negative ion model, respectively.

The metabolic profiles of QC samples are displayed in Figure 1, and the results show that the metabolic profiles of QC samples exhibit high reproducibility in both positive/negative ion modes. Principal component analysis (PCA) of serum sample metabolome is shown in Figure 2, and the results indicate that dietary treatments altered the serum metabolite profiles of rainbow trout both in positive and negative ion mode (Figure 2A,B). Moreover, PCA results of serum metabolome profiles of each treatment group versus the FM group are presented in Figure 2C–H.

### 3.2. OPLS-DA Analysis and Differential Metabolite Identification

**Figure 3:**
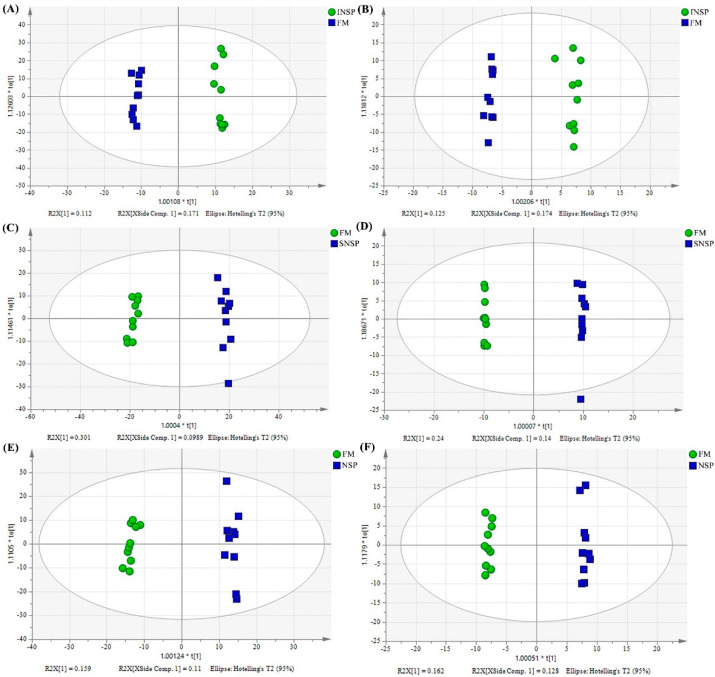
OPLS-DA analysis results between FM and different non-starch polysaccharide addition groups. (**A**,**B**) are plots of OPLS-DA scores in positive and negative ion mode between FM and INSP groups, respectively. (**C**,**D**) are plots of OPLS-DA scores in positive and negative ion mode between FM and SNSP groups, respectively. (**E**,**F**) are plots of OPLS-DA scores in positive and negative ion mode between FM and NSP groups, respectively.

OPLS-DA analysis of serum samples is presented in Figure 3. In both positive and negative ion modes, the OPLS-DA model has good accuracy and interpretation rate. Using the Variable Importance in the Projection (VIP) value of the OPLS-DA model (threshold > 1), combined with the *p*-value of the t-test (*p* < 0.05), we identified metabolites that were significantly different between the FM group and the different treatment groups under the positive and negative ion model (Supplementary Tables S2–S4). Qualitative analysis of differential metabolites was accomplished by searching an online database (Metlin, https://metlin.scripps.edu; accessed on 9 March 2022), comparing mass charge ratios (m/z) or exact molecular mass of mass spectra.

### 3.3. Metabolic Pathway Enrichment Analysis of Differential Metabolites

**Figure 4:**
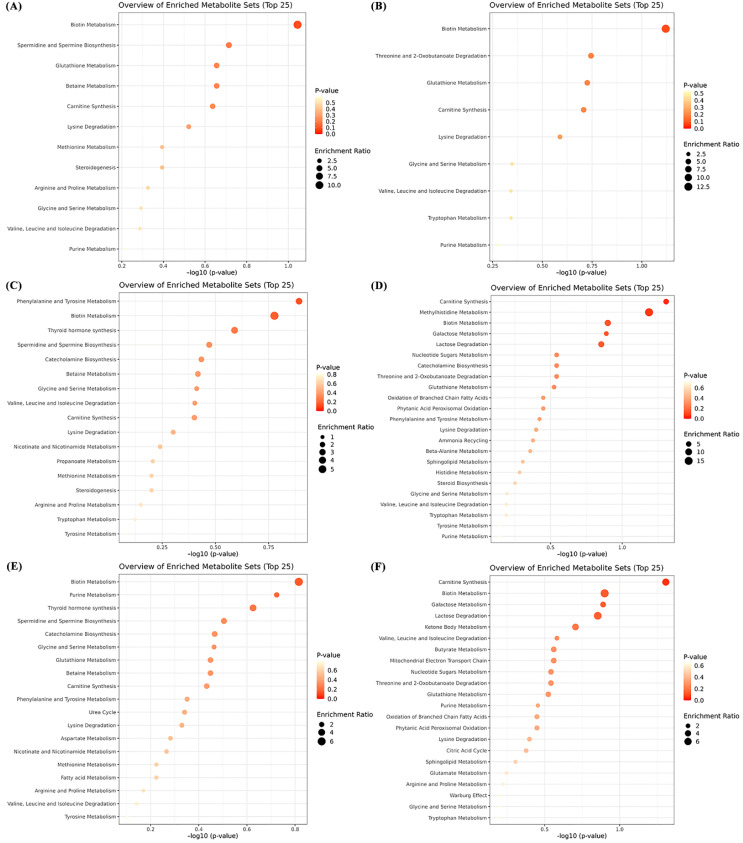
Metabolic pathway analysis of differential metabolites between FM and INSP (**A**,**B**), FM and SNSP (**C**,**D**), FM and NSP (**E**,**F**) groups. (**A**,**C**,**E**): Positive ion mode; (**B**,**D**,**F**): Negative ion mode.

To map the identified differential metabolites into the metabolic pathway, the MetaboAnalyst 5.0 online website (https://www.metaboanalyst.ca/MetaboAnalyst/home.xhtml; accessed on 20 February 2022) was utilized to perform metabolic pathway analysis (MetPA) in positive and negative ion mode between different treatment groups. Differential metabolites between FM and INSP groups were not enriched in any metabolic pathways (Figure 4A,B). In the negative ion mode, metabolites with significant differences between the FM and SNSP groups were enriched in Carnitine synthesis (*p* < 0.05). In the positive ion mode, no metabolic pathways were enriched (Figure 4C,D). Similarly, although no metabolic pathway was enriched in the positive ion mode, significantly different metabolites in the FM and NSP groups were enriched in the Carnitine synthesis in the negative ion mode (Figure 4E,F; *p* < 0.05).

### 3.4. Microbiota Structure

**Figure 5:**
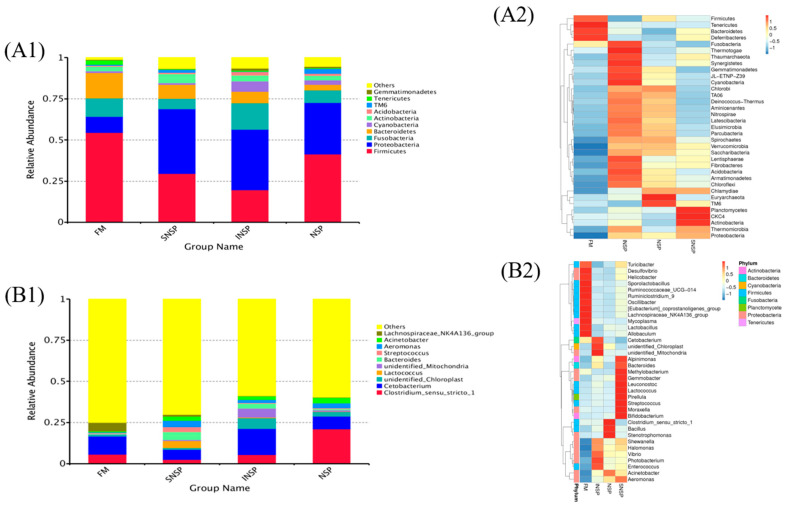
Gut microbiota structure of rainbow trout fed by test feeds. (**A1**) and (**B1**) are showed top 10 microorganisms with the highest abundance between Phylum level and genus, respectively. (**A2**) and (**B2**) are showed the top 35 genera in abundance, and clustered at the phyla level and genus level, respectively.

**Figure 6:**
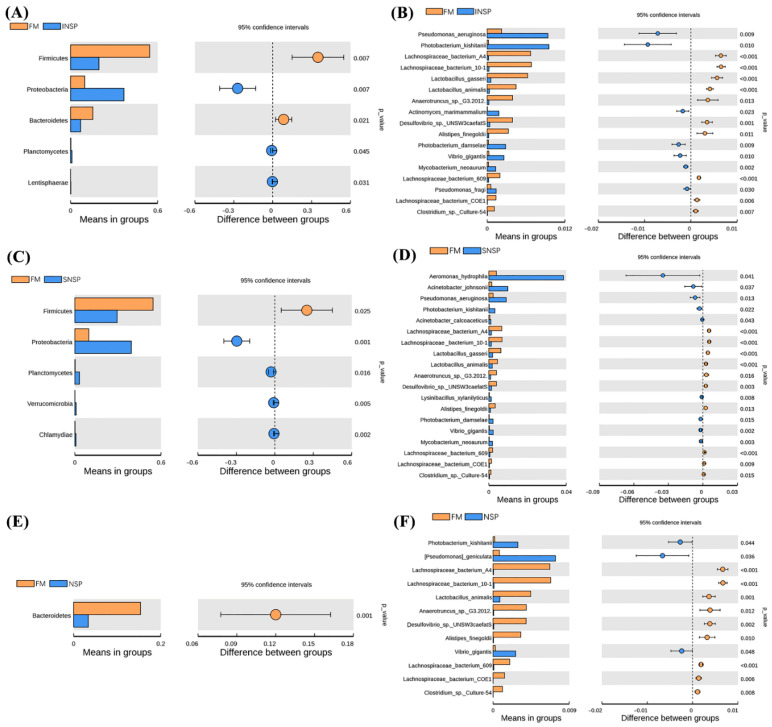
Microorganisms that were significantly different between each treatment group and the FM group. (**A**,**C**,**E**) show the differential flora between the INSP, SNSP, and NSP groups and the FM group at the phylum level, respectively, while (**B**,**D**,**F**) show them at the species level.

A total of 1,045,516 effective tags were obtained from 16 intestinal samples (Table 1), and subsequent analysis showed that test feeds had no significant influence on the Shannon, Simpson, ACE, and Chao1 indices of gut microbiota (*p* > 0.05). Anosim analysis showed that there remain significant differences in the microbiota between the treatment groups and the FM group (*p* < 0.05; Table 2). Furthermore, the composition analysis of microbiota showed that Firmicutes, Proteobacteria, Fusobacterial, and Bacteroidetes are the four dominant phylums of all groups (Figure 5(A1)), and the four dominant genus of all groups are *Clostridium sensu stricto1*, *Cetobacterium*, *Lactococcus,* and *Bacteroides* (Figure 5(B1)). Figure 5(A2,B2) shows more visual changes in the microbiota at the phylum level and genus level, respectively. Microbiota structure analysis showed that the abundance of Firmicutes in the SNSP group and the abundance of Firmicutes and Bacteroidetes in the INSP group was dramatically lower than that in the FM group, whereas the abundance of Proteobacterial, Planctomycetes, and Lentisphaerae in the SNSP and INSP groups was dramatically higher than that in the FM group (Figure 6A,C; *p* < 0.05). The abundance of Bacteroidetes in the NSP group was dramatically lower than that in the FM group (Figure 6E; *p* < 0.05). The strains with significant differences between each test group (INSP, SNSP, and NSP) and the FM group are shown in Figure 6B,D,F, respectively. Moreover, the LEfSe analysis results more intuitively present the influences of test feeds on the microbiota (Supplementary Figure S1).

## 4. Discussion

Dietary NSPs are often treated as dietary fiber, although there are some differences in their composition [23]. In our previous study, dietary NSPs showed the ability to alter the microbiota diversity and structure of fish, thereby affecting intestinal health [8,11], and dietary SNSP tends to increase the abundance of fermentable bacteria in the gut more than dietary INSP [11]. Sinha et al. [24] suggested that dietary SNSP increases chyme viscosity, prolongs transit time, and reduces gut oxygen partial pressure, which favors the proliferation of fermentable bacteria. Alpha diversity is commonly used to assess gut microbiota homeostasis and function. In this trial, dietary INSP, SNSP, or NSPs supplementation all exhibited limited influence on the microbiota Alpha diversity of rainbow trout. Similarly, dietary SNSP (pectin and xylan) supplementation had no significant influence on the microbiota Alpha diversity of yellow catfish [25], and our previous study also showed that dietary INSP, SNSP, or NSPs supplementation had limited influence on the microbiota Alpha diversity of GIFT tilapia [13]. Conversely, 3% dietary NSPs supplementation increased the microbiota Alpha diversity of largemouth bass, whereas 18% dietary NSPs supplementation showed an opposite result [8]. Moreover, 8% dietary SNSP (carboxymethyl cellulose) supplementation dramatically reduced the microbiota Alpha diversity of this species compared to 8% dietary INSP (cellulose) supplementation [11]. This evidence suggests that the influences of dietary NSPs on fish microbiota Alpha diversity are closely related to fish species.

Core flora has an important role in gut physiological function and microbiota homeostasis [26]. Composition analysis showed that Firmicutes, Proteobacteria, Fusobacterial, and Bacteroidetes were the dominant phylum in all groups, suggesting that these phyla constitute the core flora of rainbow trout, which is highly consistent with previous studies [27]. Additionally, although dietary inclusion of different NSPs types did not change the composition of core flora in this trial, it significantly altered the abundance of some phyla, such as: Both dietary INSP and SNSP inclusions induced a reduction in the abundance of Firmicutes and an increase in the abundance of Proteobacteria, and both dietary INSP and NSPs inclusions decreased the abundance of Bacteroidetes. These results suggest that dietary inclusion of different NSPs types differentially altered the physiological function and microbiota homeostasis of rainbow trout.

Proteobacteria mainly consist of *Pseudomonas aeruginosa* and *Photobacterium kishitanii* species in this trial, and these two species are widely considered pathogenic bacteria [27]. Elevated abundance of this phylum is often considered a biomarker of disorganization of the gut microbiota [28]. The abundance of these two species has increased dramatically since dietary inclusion of INSP or SNSP, and the abundance of *P. kishitanii* increased dramatically in the NSP group, suggesting that dietary NSPs inclusion disrupts gut microbiota homeostasis and is detrimental to the gut health of rainbow trout.

Firmicutes mainly consist of the *Clostridia* and *Bacilli* class, this phylum plays an essential role in maintaining fish gut health, such as the production of butyric acid for the repair and proliferation of intestinal epithelial cells [29], and the involvement in the phytosterol and cholesterol biosynthesis [27]. Hence, a decrease in the abundance of Firmicutes in the INSP and SNSP groups also suggests that dietary INSP and SNSP supplementation are detrimental to gut health.

Bacteroidetes, mainly *Alistipes finegoldii* species in this trial, have shown the ability to stimulate the growth of broilers [30]. The fermentation end-product of this strain is succinate, which is capable of providing energy to the host [31]. Moreover, succinate can be converted to propionate by numerous other Bacteroidetes bacteria, thereby benefiting gut health [30,32]. In this trial, the abundance of *A. finegoldii* decreased dramatically in all treatment groups compared to the FM group, this may be due to the significant decrease of Bacteroidetes in NSP, which reduces the production of propionate. This result suggests that dietary NSPs are detrimental to the gut health of rainbow trout.

The serum metabolome is considered the intermediate phenotype between genomic and final phenotype [33] and is widely used to systemically assess the psychological influence of dietary ingredients on aquatic animals [34,35]. In this trial, OPLS-DA analysis showed that dietary inclusion of different NSPs types caused a significant change in metabolites compared with the FM group, the specific results were presented in Supplementary Tables S2–S4. To explore the specific effects of so many differential metabolites, metabolic pathway enrichment analysis was performed on these differential metabolites. Specifically, carnitine synthesis was the only metabolic pathway that was significantly different (upregulated) in both the SNSP and NSP groups compared to in the FM group. Carnitine is essential for the fatty acid β-oxidation process, which can transport long-chain fatty acids into the mitochondria for β-oxidative catabolism through binding to acyl-CoA [36]. The absorption process of carnitine in the gut is highly correlated with gut microbiota [37]. Trimethyl lysine dioxygenase is the first enzyme in carnitine synthesis, and it requires iron as a co-factor for its activity [38]. NSPs contain a large amount of hydroxyl and carboxyl units to bind with mineral elements [39,40], and a previous study has shown that dietary NSPs supplementation accelerates iron excretion with feces [41]. Noteworthy, high SNSP diets more extremely reduced the apparent absorption coefficient of dietary iron in *Clarias gariepinus* compared to low SNSP diets [42], implying that SNSP is the dominant NSPs type accelerating iron excretion. This evidence suggests that dietary SNSP may inhibit carnitine synthesis by accelerating iron excretion and consequently disrupt carnitine metabolic homeostasis. Therefore, an upregulation in the carnitine synthesis metabolic pathway may be a response to poor carnitine activity caused by dietary SNSP supplementation (SNSP and NSP groups). Conversely, dietary INSP seems to not have this adverse impact on rainbow trout. Moreover, previous studies have shown that downregulated carnitine synthesis would inhibit fatty acid β-oxidation efficiency in zebrafish [43], and dietary SNSP tends to inhibit the dietary lipid absorption process in fish [9,24]. Hence, our results may also suggest that the upregulation of the carnitine synthesis metabolic pathway may be a physiological strategy by which fish attempt to cope with reduced lipid intake by increasing the efficiency of fatty acid β-oxidation.

## 5. Conclusions

In summary, dietary NSPs have no significant influence on gut microbiota Alpha diversity, but dramatically alter the microbiota structure. Specifically, dietary NSPs increased the abundance of pathogenic bacteria (*P. aeruginosa* and *P. kishitanii*) and decreased the abundance of beneficial microorganisms (Firmicutes and *A. finegoldii*). Besides, dietary SNSP upregulated the carnitine synthesis metabolic pathway, whereas dietary INSP did not. Our data suggest that dietary NSPs are detrimental to gut microbiota homeostasis and the health of rainbow trout, and dietary SNSP exhibit a stronger ability to interfere with physiological metabolism of rainbow trout than INSP.

## Figures and Tables

**Table 1:** Intestinal microbiota α-diversity of rainbow trout feed by test feeds.

	FM	INSP	NSP	NSPs
Effective Tags	63,957.00 ± 1300.49	64,044.00 ± 3384.14	65,748.25 ± 5820.50	67,629.75 ± 4018.80
Shannon	6.65 ± 0.25	7.36 ± 0.17	7.12 ± 1.04	6.02 ± 1.29
Simpson	0.94 ± 0.02	0.93 ± 0.03	0.97 ± 0.01	0.88 ± 0.06
Chao1	1667.05 ± 8.84	2762.44 ± 505.06	2144.58 ± 126.56	2203.73 ± 525.44
ACE	1705.18 ± 51.96	2746.35 ± 513.16	2179.36 ± 125.66	2259.24 ± 531.05
Goods coverage	0.99 ± 0.00	0.99 ± 0.00	0.99 ± 0.00	0.99 ± 0.00

**Table 2:** Anosim analysis of intestinal flora.

Group	*R*-Value	*p*-Value
FM-INSP	0.781	0.032
FM-SNSP	1.000	0.021
FM-NSP	0.583	0.028

The *R*-value was between (−1, 1); *R*-value > 0, indicating that between-group differences are greater than within-group differences, whereas *R*-value < 0 indicates that within-group differences are greater than between-group differences. *p* < 0.05 represents a significant difference.

## Data Availability

The data presented in this study are available in the main article and the Supplementary Materials.

## Supplementary Materials

The following supporting information can be downloaded at: https://www.mdpi.com/article/10.3390/metabo12121167/s1. Table S1: Diets formulation and nutrient composition used in this trial, Table S2: Differential metabolites between FM and INSP groups, Table S3: Differential metabolites between FM and SNSP groups, Table S4: Differential metabolites between FM and NSP groups. Figure S1: LEfSe analysis of microbiota in grass carp fed by test feeds.
