# Supplementary material for: Feeding Rainbow Trout with Different Types of Non-Starch Polysaccharides: Impacts on Serum Metabolome and Gut Microbiota
Source: Metabolites. 2022 Nov 23;12(12):1167. doi: 10.3390/metabo12121167 (PMC9787720; doi:10.3390/metabo12121167)
Supplement: Supplementary file 1 [file metabolites-12-01167-s001.zip › metabolites-2017248-supplementary.pdf]

Supplementary Table S1: Diets formulation and nutrient composition used in this trial.

	FM	INSP	NSP	NSPs
Ingedients				
Fish meal[1]	59.00	59.00	59.00	59.00
Wheat flour	2.75	2.75	2.75	2.75
α-starch	24.80	16.80	8.00	0.00
Cellulose	–	8.00	–	8.00
Water soluble NSP[2]	–	–	16.80	16.80
Soybean oil	10.60	10.60	10.60	10.60
Soybean lecithin	0.50	0.50	0.50	0.50
$Ca(H_2PO_4)_2$	1.00	1.00	1.00	1.00
Choline chloride (50%)[1]	0.30	0.30	0.30	0.30
Ethoxyquin (30%)[1]	0.03	0.03	0.03	0.03
Vitamin C[3]	0.02	0.02	0.02	0.02
Mineral premix[4]	0.60	0.60	0.60	0.60
Vitamin premix[5]	0.40	0.40	0.40	0.40
Nutrient composition				
Dry matter (DM, %)	90.55	90.86	93.70	93.84
Crude protein (% DM)	42.26	42.69	41.95	42.37
Crude lipid (% DM)	17.12	17.40	17.10	17.35
Ash (% DM)	12.43	12.16	12.02	11.76
Gross energy (kJ/g DM)	21.21	21.74	21.73	22.02
Viscosity (cP)	70.00	37.50	24.00	16.00

[1]Supplied by Kunming Tianyuan Feed Co., Ltd. (Yunnan, China); fish meal, 66.3% crude protein, 15.8% crude lipid.

[2]SNSP (soluble non-starch polysaccharides) including 4.8% araboxylan, 1.12% β-glucan, 1.28% mannosan, 9.60% pectin.

[3]L-Ascorbate-2-polyphosphate (35%), supplied by Galaxy Chemicals Co., Ltd. (Hubei, China).

[4]Vitamin premix (g/kg mixture): retinyl acetate (2800000 IU/g), 2; cholecalciferol, 0.03; DL-α-tocopheryl acetate, 30; menadione, 3; thiamine hydrochloride, 8; riboflavin, 11; pyridoxine hydrochloride, 8; vitamin B_{12}, 0.02; ascorbic acid, 50; folic acid, 1; biotin, 0.1; niacin, 30; calcium D-pantothenate, 32; inositol, 25.

[5]Mineral premix (g/kg mixture): $MgSO_4 \bullet 7H_2O$, 180; KI, 1; $FeSO_4 \bullet H_2O$, 260; $ZnSO_4 \bullet H_2O$, 180; $CuSO_4 \bullet 5H_2O$, 25; $Na_2Se_2O_3$, 0.01; $MnSO_4 \bullet H_2O$, 180; $CoCl_2 \bullet 6H_2O$, 0.75.

Supplementary Table S2: Differential metabolites between FM and INSP groups.

No.	VIP	Name	Molecular Weight	RT [min]	Fold INSP/FM	T test
**Positive ion model**						
1	1.75	L-Proline	115.0630	0.89	0.91	0.007
2	1.48	L-Pyroglutamic acid	129.0421	1.20	0.42	0.027
3	1.90	Vigabatrin	129.0785	0.78	0.61	0.003
4	1.83	L- (+)-Leucine	131.0941	1.42	0.38	0.004
5	1.86	DL-Lysine	146.1050	0.78	0.59	0.004
6	1.64	L- (-)-methionine	149.0505	1.12	0.51	0.013
8	1.91	Xanthine	152.0328	1.19	–0.64	0.003
9	1.41	(E)-p-coumaric acid	164.0469	1.21	0.40	0.036
10	1.41	DL-TYROSINE	181.0734	1.21	0.39	0.037
11	1.75	Zalcitabine	211.0949	1.32	0.72	0.007
12	1.35	2-Amino-9-pentofuranosyl-1,9-dihydro-6H-purin-6-one	283.0910	1.22	–0.40	0.046
13	1.63	Androstane-3,16-diol	292.2393	10.96	–0.70	0.013
14	1.55	13-KODE	294.2186	9.07	0.81	0.020
15	1.75	8-(2-Octyl-1-cyclopropen-1-yl) octanoic acid	294.2548	11.80	–0.78	0.007
16	1.49	DErySphinganine	301.2975	6.56	–0.29	0.025
17	1.61	5,6-Dihydroretinoic acid	302.2237	9.19	0.38	0.015
18	1.59	Fructoselysine	308.1574	0.77	0.36	0.016
19	1.86	Pregnane-3,20-dione	316.2391	10.69	0.52	0.004
20	2.05	loganate	376.1366	3.63	–0.45	0.001
**Negative ion model**						
1	1.87	Gamma-Hydroxybutyric acid	104.0471	1.60	0.66	0.002
2	1.41	Ethyl lactate	118.0627	3.24	0.88	0.027
3	1.93	L- (-)-Threonine	119.0581	0.85	0.88	0.001
4	1.30	L-Pyroglutamic acid	129.0423	1.21	0.33	0.044
5	1.75	(Hydroxyethyl)methacrylate	130.0627	3.76	0.51	0.004
6	1.27	L- (+)-Leucine	131.0943	1.42	0.33	0.049
7	1.73	DL-Lysine	146.1052	0.84	0.68	0.005
8	1.46	Ethyl 3-oxohexanoate	158.0939	4.43	0.50	0.021
9	1.41	2-Hydroxyoctanoic acid	160.1095	4.78	0.66	0.027
10	1.63	Pentonic acid	166.0473	0.87	–1.26	0.008
11	1.31	Uric Acid	168.0280	0.91	0.53	0.042
12	1.58	1,3,4-Trihydroxy-5-oxocyclohexanecarboxylic acid	190.0471	0.93	–0.49	0.011
13	1.91	Metirosine	195.0889	6.50	–0.21	0.001
14	1.47	DL-Tryptophan	204.0891	3.35	0.40	0.020
15	1.62	5,6-Dihydroretinoic acid	302.2233	9.19	0.31	0.009

Supplementary Table S3: Differential metabolites between FM and SNSP groups.

No.	VIP	Name	Molecular Weight	RT [min]	Fold INSP/FM	T test
**Positive ion model**						
1	1.39	Trimethylamine N-oxide	75.0681	0.88	1.77	0.000
2	1.10	Piperidine	85.0888	1.35	0.88	0.005
3	1.17	N-Methylpyrrolidone	99.0680	2.11	0.51	0.002
4	1.74	Indole	117.0575	3.41	1.05	0.000
5	1.36	L-(+)-Valine	117.0785	0.89	0.90	0.000
6	1.18	Nicotinamide	122.0476	1.19	0.53	0.002
7	1.40	Vigabatrin	129.0785	0.78	0.93	0.000
8	1.02	CREATINE	131.0691	0.89	1.65	0.011
9	1.64	L-(+)-Leucine	131.0941	1.42	0.84	0.000
10	1.51	Naphthalen-2-amine	143.0731	3.36	0.79	0.000
11	1.43	DL-Lysine	146.1050	0.78	0.96	0.000
12	1.32	L-(-)-methionine	149.0505	1.12	0.94	0.000
13	1.30	(E)-p-coumaric acid	164.0469	1.21	0.81	0.000
14	1.34	DL-Phenylalanine	165.0784	2.01	0.66	0.000
15	1.32	DL-TYROSINE	181.0734	1.21	0.83	0.000
16	1.30	4-amino-2-hydroxyamino-6-nitrotoluene	183.0644	7.97	–2.81	0.000
17	1.17	DL-Tryptophan	204.0893	3.33	0.42	0.002
18	1.32	Sinapyl alcohol	210.0890	3.88	2.15	0.000
19	1.14	primidone	218.1048	3.76	1.03	0.003
20	1.10	3-(1H-Imidazol-4-yl)-2-oxopropyl dihydrogen phosphate	220.0251	17.16	0.76	0.005
21	1.40	threonylphenylalanine	266.1257	4.00	3.39	0.000
22	1.37	Androstane-3,16-diol	292.2393	11.03	–1.43	0.000
23	1.50	13-KODE	294.2186	9.07	1.27	0.000
24	1.24	8-(2-Octyl-1-cyclopropen-1-yl) octanoic acid	294.2548	11.80	–1.11	0.001
25	1.30	2-aminooctadec-4-ene-1,3-diol	299.2817	6.96	2.22	0.000
26	1.49	DErySphinganine	301.2974	6.61	1.08	0.000
27	1.34	5,6-Dihydroretinoic acid	302.2237	9.19	–0.79	0.000
28	1.14	Fructoselysine	308.1574	0.77	–0.44	0.003
29	1.18	Pregnane-3,20-dione	316.2391	10.69	–0.60	0.002
30	1.46	Phytosphingosine	317.2921	5.65	–0.62	0.000
31	1.37	Allopregnanolone	318.2548	11.29	–1.49	0.000
32	1.50	Adipostatin A	320.2705	12.09	–1.91	0.000
33	1.61	Retinyl acetate	328.2392	9.62	–1.12	0.000
34	1.48	(2R)-2-Hydroxy-3-[(6Z,9Z,12Z,15Z)-6,9,12,15-octadeca tetraenoyloxy] propyl 2-(trimethylammonio)ethyl phosphate	515.2990	6.61	–0.73	0.000
**Negative ion model**						
1	1.24	Gamma-Hydroxybutyric acid	104.0471	1.60	0.79	0.005
2	1.32	Ethyl lactate	118.0627	3.24	1.90	0.002
3	1.63	L-(-)-Threonine	119.0581	0.85	1.14	0.000
4	1.29	L-Pyroglutamic acid	129.0423	1.21	0.56	0.003
5	1.21	(Hydroxyethyl)methacrylate	130.0627	3.76	0.85	0.006
6	1.73	L-(+)-Leucine	131.0943	1.42	0.89	0.000
7	1.70	2,3,4-Trihydroxybutanoic acid	136.0369	0.88	1.60	0.000
8	1.76	DL-Lysine	146.1052	0.84	2.17	0.000
9	1.02	2,4-Dideoxy-3-C-methylpentonic acid	148.0732	1.30	0.62	0.025
10	1.91	2,3,4,5-Tetrahydroxypentanal	150.0525	0.90	4.22	0.000
11	1.94	pentane-1,2,3,4,5-pentol	152.0682	0.85	5.01	0.000
12	1.05	DL-Histidine	155.0691	0.85	0.36	0.021
13	1.11	DL-Phenylalanine	165.0785	2.01	0.49	0.013
14	1.98	Pentonic acid	166.0473	0.87	6.18	0.000
15	1.08	veratraldehyde	166.0625	3.99	1.19	0.016
16	1.15	Uric Acid	168.0278	1.17	0.89	0.010
17	1.02	Vitamin C	176.0316	0.91	4.24	0.024
18	1.08	Hexose	180.0629	0.90	1.22	0.017
19	1.50	Hexitol	182.0785	0.84	5.42	0.000
20	1.40	DL-Tryptophan	204.0891	3.35	0.62	0.001
21	1.20	5,6-Dihydroretinoic acid	302.2233	9.19	–0.38	0.007
22	1.84	Melilotoside	326.1007	0.88	7.07	0.000
23	1.79	Chlorogenic acid	354.0954	0.88	5.66	0.000
24	1.68	Gentiopicroside	356.1109	0.88	5.75	0.000

Supplementary Table S4: Differential metabolites between FM and NSP groups.

No.	VIP	Name	Molecular Weight	RT [min]	Fold INSP/FM	T test
**Positive ion model**						
1	1.25	Trimethylamine N-oxide	75.0681	0.88	1.28	0.026
2	1.36	Indole	117.0575	3.41	0.25	0.014
3	1.33	Nicotinamide	122.0476	1.19	0.44	0.017
4	1.50	L-Pyroglutamic acid	129.0421	1.20	0.44	0.005
5	1.84	Vigabatrin	129.0785	0.78	0.84	0.000
6	1.80	L-(+)-Leucine	131.0941	1.42	0.58	0.000
7	1.23	Naphthalen-2-amine	143.0731	3.36	0.35	0.029
8	1.82	DL-Lysine	146.1050	0.78	0.85	0.000
9	1.21	L-(-)-methionine	149.0506	1.19	0.47	0.032
10	1.31	Guanine	151.0489	1.22	–0.65	0.019
11	1.52	Xanthine	152.0328	1.19	–0.69	0.005
12	1.33	(E)-p-coumaric acid	164.0469	1.21	0.55	0.017
13	1.26	Uric Acid	168.0278	1.17	0.94	0.025
14	1.40	DL-Arginine	174.1112	0.86	0.61	0.011
15	1.34	DL-TYROSINE	181.0734	1.21	0.55	0.016
16	1.48	Sinapyl alcohol	210.0890	3.88	1.09	0.007
17	1.17	primidone	218.1048	3.76	0.72	0.039
18	1.45	3-(1H-Imidazol-4-yl)-2-oxopropyl dihydrogen phosphate	220.0249	17.38	0.78	0.008
19	1.57	threonylphenylalanine	266.1257	4.00	3.42	0.003
20	1.23	Arabinosylhypoxanthine	268.0801	1.22	0.28	0.029
21	1.42	2-Amino-9-pentofuranosyl-1,9-dihydro-6H-purin-6-one	283.0910	1.22	–0.72	0.010
22	1.93	13-KODE	294.2186	9.07	1.43	0.000
23	1.18	2-aminooctadec-4-ene-1,3-diol	299.2817	6.96	0.42	0.038
24	1.34	Fructoselysine	308.1574	0.77	–0.39	0.016
25	1.33	Adipostatin A	320.2705	12.09	–0.95	0.017
26	1.21	L-Palmitoylcarnitine	399.3333	7.74	1.61	0.032
**Negative ion model**						
1	1.94	Gamma-Hydroxybutyric acid	104.0471	1.60	0.87	0.000
2	1.21	Succinic acid	118.0264	1.30	0.68	0.029
3	1.66	Ethyl lactate	118.0627	3.24	1.54	0.001
4	1.92	L-(-)-Threonine	119.0581	0.85	1.45	0.000
5	1.66	L-Pyroglutamic acid	129.0423	1.21	0.47	0.001
6	2.06	(Hydroxyethyl)methacrylate	130.0627	3.76	1.11	0.000
7	2.10	L-(+)-Leucine	131.0943	1.42	0.82	0.000
8	2.15	2,3,4-Trihydroxybutanoic acid	136.0369	0.88	1.44	0.000
9	1.27	Hypoxanthine	136.0383	1.22	0.31	0.021
10	1.88	DL-Lysine	146.1052	0.84	1.94	0.000
11	2.27	2,3,4,5-Tetrahydroxypentanal	150.0525	0.90	3.91	0.000
12	2.33	pentane-1,2,3,4,5-pentol	152.0682	0.85	4.93	0.000
13	1.30	2-Hydroxyoctanoic acid	160.1095	4.78	0.62	0.018
14	2.31	Pentonic acid	166.0473	0.87	6.32	0.000
15	1.14	veratraldehyde	166.0625	3.99	0.77	0.043
16	1.42	Uric Acid	168.0278	1.17	1.09	0.009
17	1.25	Hexose	180.0629	0.90	0.74	0.024
18	1.76	Hexitol	182.0785	0.84	4.29	0.000
19	1.17	DL-Tryptophan	204.0891	3.35	0.39	0.036
20	1.60	2-(Hexopyranosyloxy)-3-hydroxypropanoic acid	268.0797	1.23	0.40	0.002
21	1.47	Melilotoside	326.1007	0.88	6.28	0.006
22	1.49	Retinyl acetate	328.2389	7.44	0.29	0.005
23	1.53	Chlorogenic acid	354.0954	0.88	5.00	0.004
24	1.37	Gentiopicroside	356.1109	0.88	4.17	0.012

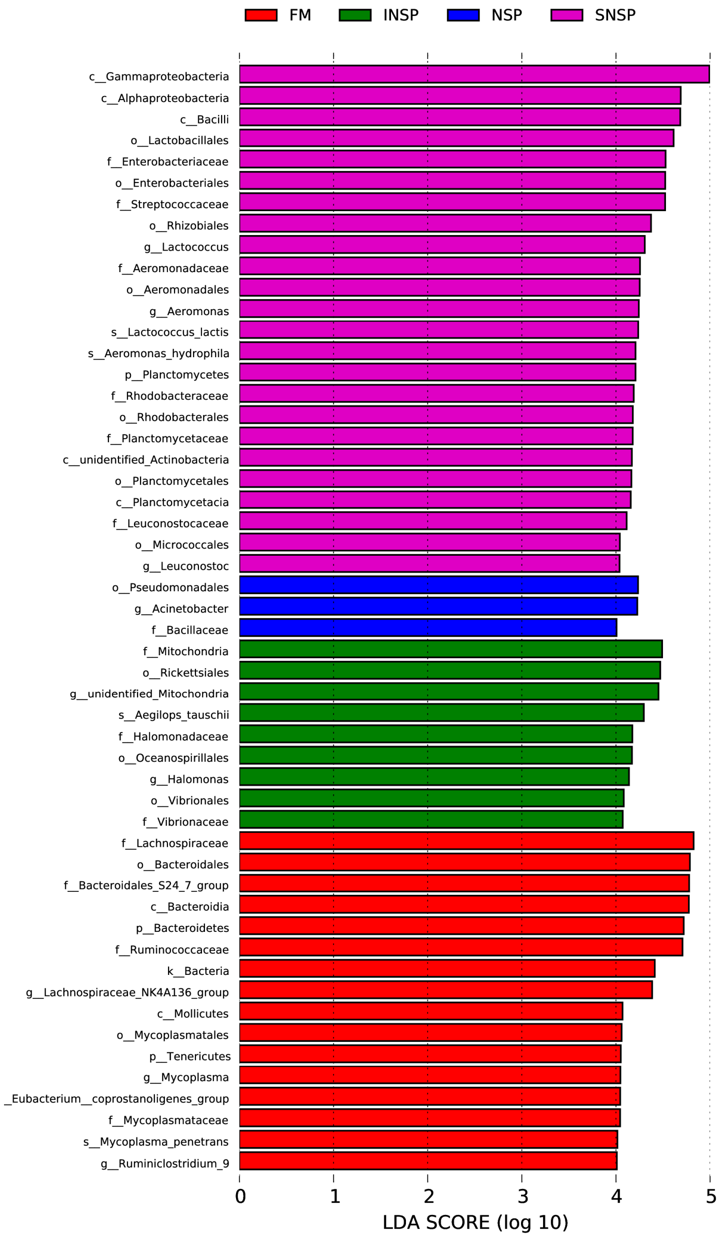

Supplementary Figure S1: LEfSe analysis of microbiota in grass carp fed by test feeds.
